# Composition Design and Fundamental Properties of Ultra-High-Performance Concrete Based on a Modified Fuller Distribution Model

**DOI:** 10.3390/ma16020700

**Published:** 2023-01-11

**Authors:** Xiaogeng Xie, Junqi Fan, Peng Guo, Haoliang Huang, Jie Hu, Jiangxiong Wei

**Affiliations:** 1 School of Materials Science and Engineering, South China University of Technology, Guangzhou 510640, China; 2 Research Institute for National Defense Engineering of Academy of Military Sciences PLA China, Luoyang 471023, China

**Keywords:** modified Fuller distribution model, UHPC, mixture proportion design, mechanical property, durability

## Abstract

Both the discrete and continuous particle packing models are used to design UHPC, but the influences of a water film covering the particle surfaces on the compactness of the particle system were not considered in these models. In fact, the water film results in a certain distance between solid particles (DSP), which affects the compactness of the particle system, especially for cementitious materials with small particle sizes. In the present study, the mixture design method for UHPC was proposed based on the Fuller distribution model modified using the DSP. Then, the components of cementitious materials and aggregates were optimized, and the UHPC matrices with high solid concentrations were obtained. The results showed that the solid concentration, slump flow, and compressive strength of the UHPC matrix reached 77.1 vol.%, 810 mm, and 162.0 MPa, respectively. By replacing granulated blast furnace slag (GBFS) with quartz powder (QP), the flexural strength of the UHPC matrix was increased without reducing its compressive strength. When the steel fiber with a volume fraction of 1.5% was used, the slump flow, compressive strength, tensile strength, and flexural strength of the UHPC reached 740 mm, 175.6 MPa, 9.7 MPa, and 22.8 MPa, respectively. After 500 freeze–thaw cycles or 60 dry–wet cycles under sulfate erosion, the mechanical properties did not deteriorate. The chloride diffusion coefficients in UHPCs were lower than 3.0 × 10^−14^ m^2^/s, and the carbonation depth of each UHPC was 0 mm after carbonization for 28 days. The UHPCs presented ideal workability, mechanical properties, and durability, demonstrating the validity of the method proposed for UHPC design.

## 1. Introduction

Ultra-high-performance concrete (UHPC) has a dense microstructure due to the close packing of powder particles and the hydration of cementitious materials [1]. The tight particle packing can facilitate denser microstructure, thereby improving the mechanical properties of UHPC [2,3]. Soliman confirmed that the compressive strength of the UHPC matrix could be enhanced with the increase in matrix density and established the relationship between the compressive strength and density of the UHPC matrix [4]. Therefore, the close packing of particles is the design principle of UHPC [5].

By far, the close packing of particles in the UHPC matrix is usually achieved by experimentation or theoretical models. However, heavy trial experiments are needed to determine the optimal mixture proportion of UHPC with many kinds of components [6]. To design UHPC scientifically and efficiently, many theoretical models were proposed and then developed. These models can be divided into discrete and continuous models according to the distribution characteristics of particles [6]. The research objects of the discrete models, such as the compressible packing model (CPM) [7] and compaction–interaction packing model (CIPM) [8], are the particles with specific sizes. As a result, these models are strict with the granularity range of particles. They only use the particle size to assess the compactness of the particle system, which limits the design of cementitious materials with continuous particle size distributions [9]. The research objects of the continuous models proposed by Fuller [10], Andreasen, and Andersen [11] are the particles in a specific granularity range. The principle of continuous models is to make the particle distribution designed close to the ideal particle distribution by adjusting the size and proportion of the raw materials [10,11]. Based on the Andreasen and Andersen (A&A) particle packing model, a modified A&A (MAA) model was proposed by introducing the parameter of minimum particle size [12] and then widely employed for the mixture proportion design of UHPC [13,14]. Nevertheless, the above models only consider the packing of the solid particles in a dry condition, and the influence of the water layer covering the surface of the particles on the compactness of the solid particle system should be taken into account. In fact, the water layer results in a specific spacing between solid particles, which cannot be ignored for cementitious materials with small particle sizes [15,16,17].

Considering the influence of a water film on the packing of the solid particles suspended in a wet condition [15], the average thickness of the water film (AWFT) covering the solid particles was predicted using the D-optimal design approach [16]. Then, the new particle size (*d_new_*) was defined as the sum of the original particle size and double the AWFT [16]. By combining the modified A&A and established AWFT models, the water film was factored into the method for UHPC mixture design. However, it is worth noting that the thickness of the water film covering the particle surface is related to the size and surface properties of the solid particles [17]. Compared with the finer particles, the water film covering the coarser particles is thicker [17]. This shows that the thickness of the water film covering the finer particles could be overestimated. In comparison, the thickness of the water film covering the coarser particles could be underestimated when the AWFT was employed. According to the reported literature, the matrix accounts for 50% to 75% of the total volume of UHPC, and the median particle size of the aggregate is more than ten times that of cementitious material, implying that the aggregates are suspended in the matrix [9,14,16,18,19,20,21,22]. It can be seen that the packing of cementitious material particles, especially ultrafine powder particles like silica fume, has a significant effect on the compactness of the UHPC matrix. Therefore, it is more reasonable to consider that a different water film thickness is adsorbed onto the surface of the cementitious particles with different particle sizes in the theoretical models.

Because the particles were not in contact in the wet condition, a parameter called particle spacing was defined and deduced. The particle spacing is connected to the diameters of solid particles and then used to modify the Fuller distribution model for the design of blended cement [17]. In this study, the modified Fuller distribution model was used to optimize the cementitious material systems, and the aggregate system was also optimized by employing the Fuller distribution model. To assess the reliability of the method proposed for the mixture proportion design of UHPC, a series of UHPC matrices with high solid concentrations were designed. Then, the effects of matrix composition, such as cementitious material components, aggregate-to-cementitious material ratio (A/C), and water-to-cementitious material ratio (W/C), on the mechanical properties of UHPC matrices were investigated under similar solid concentrations. The influences of matrix composition and fiber content on the mechanical properties of UHPC were analyzed, and the effect of matrix composition on the durability performance of UHPC was discussed. Based on the mixture proportion design method proposed, the composition of the UHPC matrix could be optimized for the selected cementitious material and aggregate system. Furthermore, the cementitious material and aggregate systems component can also be adjusted according to the solid concentration of the UHPC matrix, which can provide a new way to select raw material for the mixture proportion design of UHPC.

## 2. Materials and Methods

### 2.1. Materials

**Figure 1:**
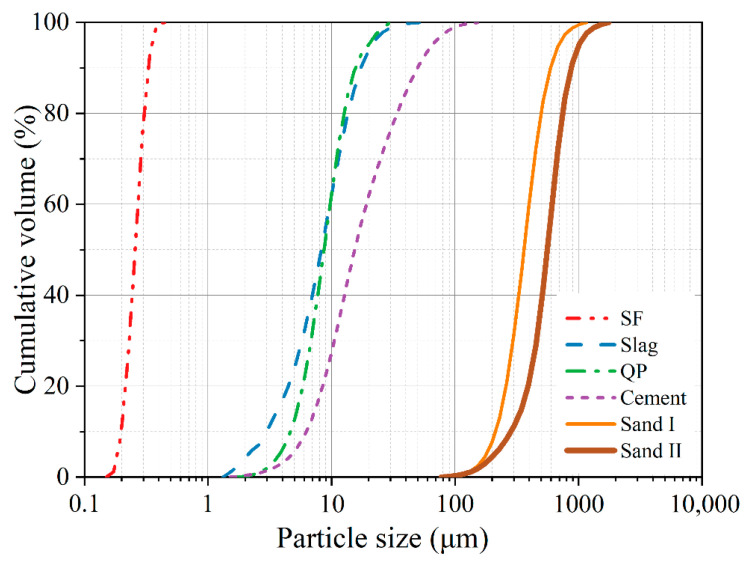
Particle size distribution of materials used.

Type II Portland cement, granulated blast furnace slag (GBFS), and densified silica fume (SF) were used as cementitious materials, and a superfine quartz powder (QP) was used as filler to replace cementitious materials. Two types of quartz sand with different size ranges were selected as fine aggregates. The chemical compositions and particle size distribution of particles are summarized in Table 1 and Figure 1. The physical properties and particle size statistics of the above materials are listed in Table 2. A polycarboxylic acid superplasticizer (SP) with a solid content of 33.0% was used to adjust the workability of the UHPC matrix. Steel fiber with a length of 13 mm and a diameter of 0.2 mm was used to prepare fiber-reinforced UHPC.

### 2.2. Experimental Methods

#### 2.2.1. Preparation of the UHPC

The fresh UHPC was prepared using a three-step feeding method. Specifically, all of the quartz sand and steel fibers were mixed for 2 min at a rate of 40 r/min to disperse the fiber via the impact force of the quartz sand. Then, the cementitious materials were added to the mixture and mixed for another 2 min at a rate of 40 r/min. In the third step, the water and SP were added to the mixture simultaneously and then mixed at a rate of 60 r/min until the fresh UHPC matrix showed ideal flowability. The first step will be omitted when preparing the UHPC matrix, which does not use steel fiber.

The fresh UHPC samples were cast into the molds whose size depended on the testing standard. Then, 30 s of vibration was applied to eliminate air voids in the fresh UHPC, and the mixture above the mold was removed. After curing for 24 h at 20 ± 1 °C and 95% relative humidity, the specimens were demolded. For the mechanical properties test, the specimens were cured with heat at 80 °C for 48 h, and the specimens for the durability test were cured in lime-saturated water at 20 ± 1 °C for 28 days.

#### 2.2.2. Solid Concentration of the UHPC Matrix

To measure the solid concentration of the UHPC matrix, the fresh UHPC matrix was prepared and then poured into a metal container with known mass. Then, 30 s of vibration was applied to eliminate air voids in the UHPC matrix. After plastering and cleaning the matrix on the container surface, the total mass of the container and the matrix in it can be obtained. The solid concentration (φ) of UHPC can be calculated using Equation (1):

(1) $$\varphi =\frac{\sum_{i\in X}V_{i}}{V_{0}}$$

where X is the set of solid particle types; $V_{0}$ is the volume of the container; and $V_{i}$ is the solid volume of type *i* particles in the container, which can be calculated using Equation (2):

(2) $$V_{i}=\frac{m_{i}}{\rho_{i}}=m_{c}\cdot \frac{R_{i/c}}{\rho_{i}}$$

where $m_{i}$ is the mass of type *i* particles in the container; $\rho_{i}$ is the specific density of type *i* particles; $R_{i/c}$ is the mass ratio of type *i* particles to cement particles, which can be determined using the mixture of UHPC matrix; and $m_{c}$ is the mass of cement particles in the container, which can be calculated using Equation (3):

(3) $$m_{c}=\frac{m_{2}-m_{1}}{1+\sum_{j\in Y}R_{j/c}}$$

where $m_{1}$ and $m_{2}$ are the mass of the container and the mass of the container filled with UHPC matrix, respectively; Y is the set of raw material types, which contains solid particles, water, and SP; and $R_{j/c}$ is the mass ratio of type *j* material to cement particles, which can be determined using the mixture of UHPC matrix. For each UHPC matrix, the average value of three duplicates was recorded as the solid concentration of the UHPC matrix.

#### 2.2.3. Workability of the UHPC

The workability of UHPC was evaluated using the slump flow of UHPC according to Chinese Standard GB/T 50080-2016 [23]. The maximum slump flow diameter and the diameter vertical with the direction of the maximum slump flow diameter were recorded after 50 s for the extension of the fresh UHPC on the steel plate. The average value of the two diameters was calculated as the slump flow of UHPC.

#### 2.2.4. Mechanical Properties of the UHPC

The compressive strength and flexural strength of UHPC were measured using specimens with sizes of 100 mm × 100 mm × 100 mm and 100 mm × 100 mm × 400 mm, respectively, according to Chinese National Standard GB/T 50081-2019 [24]. The compression testing was carried out under a loading rate of 0.8 MPa/s until the fracture of the specimen. The compressive strength of each specimen was calculated, and the average value of six duplicates was recorded as the compressive strength of the UHPC. Four-point flexural load tests were performed on six parallel specimens of each UHPC under a loading rate of 0.09 MPa/s. The average value of the flexural strength calculated was recorded as the flexural strength of the UHPC.

The tensile strength of UHPC was tested on dog bone specimens with a cross-section of 50 mm × 50 mm under direct tension based on Chinese Standard T/CBMF37-2018 [25]. To examine the eccentricity of the specimens under tension, the strain gauges were glued at the mid-point of the specimen along its axis, except for the casting surface of the specimen. Then, the strains were recorded when a direct tensile load of 2.0 kN was preloaded, and the eccentricity of the specimen was calculated according to GB/T 50081-2019 [24]. As the eccentricity was lower than 15%, the direct tensile test was carried out under a loading rate of 0.1 MPa/s. Then, a specimen was considered valid if the major fracture emerged at the zone with the cross-section of 50 mm × 50 mm. The tensile strength of the valid specimen was calculated based on the maximum load and the cross-sectional area. The average value of six duplicates was recorded as the tensile strength of the UHPCs.

#### 2.2.5. Durability of the UHPC

The durability of UHPC, such as freeze–thaw resistance, sulfate attack resistance, chloride penetration resistance, and carbonation resistance, were characterized according to Chinese National Standard GB/T 50082-2009 [26].

For the freeze–thaw resistance of UHPC, three parallel specimens with a size of 100 mm × 100 mm × 400 mm were used to determine the relative dynamic elasticity modulus and the mass loss of the UHPC after freeze–thaw cycles.

The percentage of compressive strength of UHPC after the dry–wet cycle under sulfate erosion to the original value of UHPC under standard curing was used to assess the sulfate attack resistance of UHPC. The specimens with the size of 100 mm × 100 mm × 100 mm were used for the compressive strength test. Three specimens for standard curing and three other specimens for sulfate erosion were utilized for each UHPC mixture.

To evaluate the chloride ion permeability of the UHPC, the chloride diffusion coefficients of UHPC were determined using the rapid chloride migration (RCM) test. The cylindrical specimens with a size of 100 mm in diameter and 50 mm in height were cut from the specimens with a length of 100 mm in height after curing for 21 days. The cut faces of three parallel specimens were polished and then cured in lime-saturated water for 28 days.

The carbonization depth was used as the index to evaluate the carbonation resistance of UHPC. Three parallel specimens with the size of 100 mm × 100 mm × 400 mm were used for the carbonization depth test of UHPC.

## 3. Design of the UHPC Based on the Modified Fuller Distribution Model

### 3.1. Design of the Cementitious Materials

Cementitious material is the main component of the UHPC matrix. Thus, the compactness and the flowability of the UHPC matrix are affected by the type and particle size distribution of the cementitious materials. Due to the low W/C of a UHPC matrix and low hydration degree of the cementitious materials, a cementitious material system in the UHPC matrix could be regarded as a low-activity system. According to the particle size distribution of cementitious materials used, the composition of cementitious materials in UHPC can be optimized according to the Fuller distribution model [27,28]:

(4) $$U\left(x\right)=100{\left(\frac{x}{D}\right)}^{q}$$

where U(x) is the cumulative volume of particles under x μm (%); *D* is the maximum diameter of particles in the particle system (μm); and q is the particle distribution coefficient, which can be taken as 0.4 for cementitious materials [27,28].

Considering that the particles in cement paste are not in contact, a parameter called particle spacing was defined and deduced by Zhang et al. [17], and then used to modify the Fuller distribution model. The modified Fuller distribution model is more consistent with the packing of particles in the cement paste, which can be expressed as

(5) $$\phi \left(d\right)=100\cdot {\left(\frac{\lambda D+C}{D}\right)}^{3}\cdot {\left(\frac{d}{\lambda d+C}\right)}^{3}\cdot {\left(\frac{\lambda d+C}{\lambda D+C}\right)}^{0.4}$$

where ϕ(d) is the cumulative volume of solid particles under d μm (%); *D* is the maximum diameter of solid particles in the particle system (μm); and λ and C are constants, which are 1.146 and 0.502, respectively [17].

Based on the modified Fuller distribution model, the cementitious materials component can be adjusted to make the particle distribution designed close to the ideal particle distribution. Specifically, the particle distribution range of the selected cementitious material system can be divided into several subintervals. Then, the theoretical volume of particles in each subinterval can be calculated using Equation (5). For a given cementitious material system, the sum of squares of residuals (*RSS*) between the theoretical volume and the designed volume of each subinterval can also be calculated using Equation (6). When the *RSS* is minimized by adjusting the size and proportion of the particles, the cementitious materials component can be determined:

(6) $$RSS=\sum_{i=1}^{n}{\left(V_{D}^{i}-V_{T}^{i}\right)}^{2}$$

where *n* is the number of the subinterval, $V_{D}^{i}$ is the designed particle volume in interval *i*, and $V_{T}^{i}$ is the theoretical particle volume in interval *i*.

For a particle system formed by N kinds of cementitious materials, the particle distribution range can be defined as [0, $D_{95}^{Cmax}$], where $D_{95}^{Cmax}\;$ is determined using Equation (7):

(7) $$D_{95}^{Cmax}=\max\left(D_{95}^{C_{1}},D_{95}^{C_{2}},\;\ldots ,D_{95}^{C_{N}}\right)$$

where *C_N_* is the symbol for cementitious material *N*; $D_{95}^{C_{N}}$ is the particle size when the cumulative volume of particle *N* reaches 95%.

For a binary cementitious material system formed by particles C_1_ and C_2_, this system is named C_1_-C_2_ if the median particle size of C_2_ is larger than that of C_1_. If the particle range distribution of the two cementitious materials does not overlap, the particle distribution range of C_1_-C_2_ is divided into subinterval I and subinterval II according to the minimum particle size value of C_2_. If the particle distribution range of C_1_ and C_2_ overlaps, the particle distribution range of C_1_-C_2_ is divided into two subintervals based on the median diameter of C_1_.

For a ternary cementitious material system formed by particles C_1_, C_2_, and C_3_, the system can be regarded as a binary cementitious material system formed by particles C_1_ and C_2-3_ if the median diameter of particles C_1_, C_2_, and C_3_ increase in turn. Then, the particle distribution range of C_1_-C_2_-C_3_ is divided into subinterval I and subinterval II-III by applying the method for the binary cementitious material system. The subinterval II-III is then divided into subinterval II and subinterval III. According to the above process, the subintervals can be determined for other cementitious material systems.

**Figure 2:**
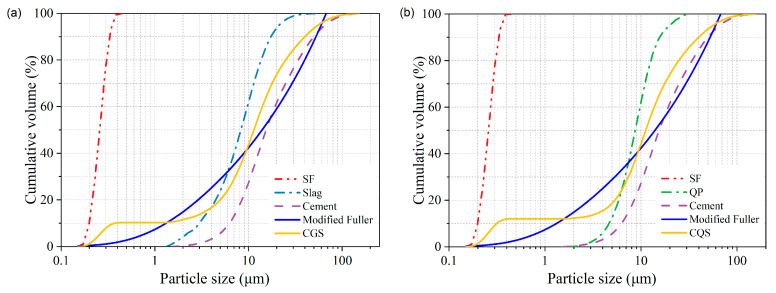
Particle size distribution of cementitious material: (**a**) CGS system; (**b**) CQS system.

Based on the modified Fuller distribution model, a CGS cementitious material system was designed using cement, GBFS, and SF. Then, the particle distribution range of CGS was divided into three subintervals, which were 0–1.73 μm, 1.73–8.82 μm, and 8.82–67.52 μm. The results showed that the *RSS* was minimized when the volumes of cement, GBFS, and SF accounted for 64.4%, 25.2%, and 10.4%, respectively. To investigate the influence of cementitious material components on the properties of the UHPC matrix under similar solid contents, a CQS cementitious material system was also designed by replacing GBFS with QP. The compositions and the particle size distributions of cementitious material systems are shown in Table 3 and Figure 2, respectively.

### 3.2. Design of the Aggregates

Since the average particle size of the aggregate was much larger than the thickness of the water film coating the aggregate particles, the effect of the water film thickness on the packing of aggregate particles could be ignored. Therefore, the composition of the aggregate system could be adjusted to make the particle distribution designed close to the ideal particle distribution by Equation (4), and the particle distribution coefficient q could be taken as 0.5 [10]. For a particle system formed by M kinds of aggregates, the particle distribution range can be defined as [$D_{10}^{Amin}$, $D_{95}^{Amax}$]. The $D_{10}^{Amin}$ and $D_{95}^{Amax}$ were determined using Equation (8) and Equation (9), respectively:

(8) $$D_{10}^{Amin}=\min\left(D_{10}^{A_{1}},D_{10}^{A_{2}},\;\ldots ,D_{10}^{A_{M}}\right)$$

(9) $$D_{95}^{Amax}=\max\left(D_{95}^{A_{1}},D_{95}^{A_{2}},\;\ldots ,D_{95}^{A_{M}}\right)$$

where *A_M_* is the symbol for aggregate *M*; $D_{10}^{A_{M}}$ and $D_{95}^{A_{M}}$ are the particle size when the cumulative volume of particle M reaches 10% and 95%, respectively.

**Figure 3:**
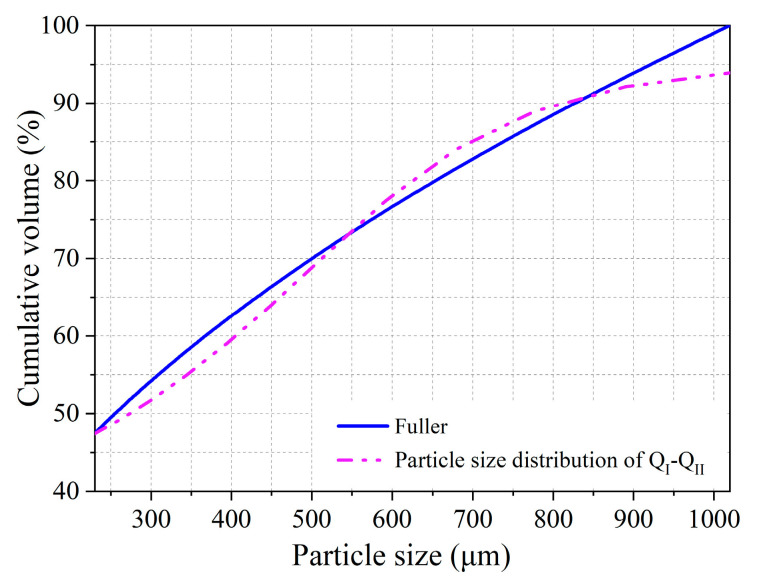
Particle size distribution of the aggregate system.

The subintervals and the composition of aggregate systems were determined according to the method for the cementitious material system. For the binary aggregate system formed by particles Q_I_ and Q_II_, the particle distribution range of this system was divided into two subintervals, which were 229.08–394.24 μm and 394.24–1019.50 μm. The results showed that the *RSS* was minimized, which can be seen as visualized in Figure 3 when the volumes of Q_I_ and Q_II_ accounted for 25.0% and 75.0%, respectively.

### 3.3. Design of the Solid Particle Mixtures

Based on the cementitious materials and aggregate systems obtained above, three mixtures for UHPC matrix preparation were designed and are listed in Table 4. The fresh UHPC matrices were prepared with a W/C of 0.16, and the solid concentration of the UHPC matrices was determined according to Section 2.2.2. To reduce the effect of matrix fluidity on the solid concentration test for UHPC matrices, the slump flow of UHPC matrices was designed between 770 ± 50 mm by adjusting the dosage of SP. As shown in Table 4, the solid concentrations of the UHPC matrix designed in this study reached 78.40%, and the solid concentration can be further improved by decreasing the W/C and increasing the A/C [29]. In the available literature, the maximum solid concentration of the UHPC matrix designed ranged from 75.8% to 83.2% [9,29,30], which demonstrated the validity of the design method proposed for UHPC with a high solid concentration.

## 4. Mechanical Properties of the UHPC Matrix

Based on the solid particle mixtures obtained above, three series UHPC matrices were designed to investigate the effects of the cementitious material composition, aggregate-to-cementitious materials ratio (A/C), and water-to-cementitious material ratio (W/C) on the compressive strength and flexural strength of UHPC matrix, as shown in Table 5. To ensure similar workability for each UHPC matrix, the slump flow of UHPC matrices was designed to be between 770 ± 50 mm by adjusting the dosage of SP.

### 4.1. The Compressive Strength of the UHPC Matrix

**Figure 4:**
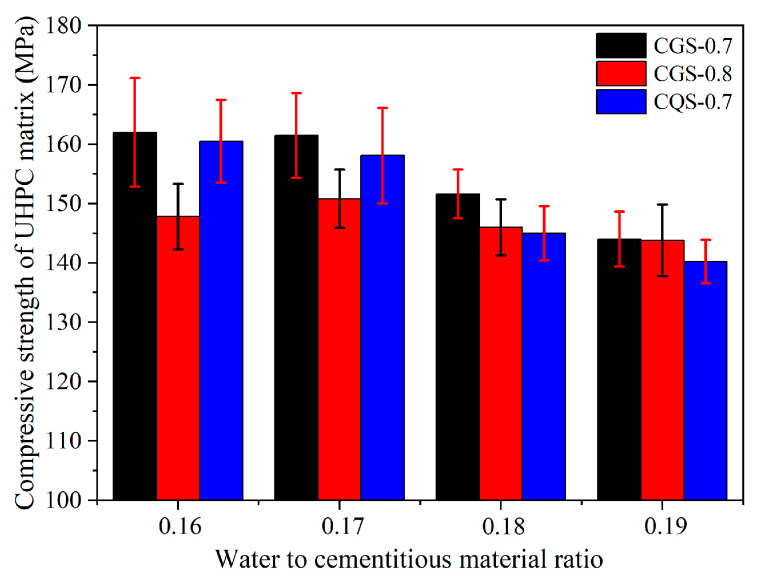
The compressive strength of the UHPC matrix.

**Figure 5:**
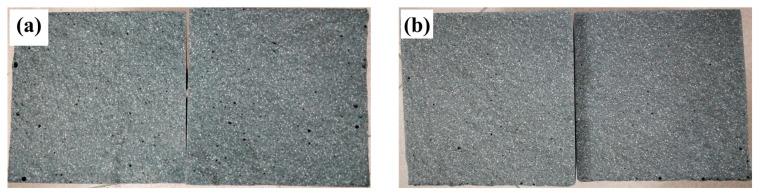
The pictures of the UHPC matrix section: (**a**) CGS-0.8-0.16; (**b**) CGS-0.8-0.17.

The compressive strength of the UHPC matrix is shown in Figure 4. For the CGS-0.7 and CQS-0.7 series, the compressive strength of the UHPC matrix decreased slightly and then decreased remarkably when the W/C increased from 0.16 to 0.19. For instance, when the W/C increased from 0.16 to 0.17, the compressive strength of the UHPC matrix for the CGS-0.7 series was decreased by only 0.5 MPa, but it dropped by 9.9 MPa when the W/C was reduced to 0.18. For the CGS-0.8 series, the compressive strength of the UHPC matrix increased from 147.8 MPa to 150.8 MPa when the W/C increased from 0.16 to 0.17. When the W/C was further reduced to 0.18, the compressive strength of the UHPC matrix was reduced to 146.0 MPa. This was mainly due to the viscosity of the UHPC paste increasing when the W/C decreased from 0.17 to 0.16, which was not conducive to the elimination of bubbles in the matrix, resulting in more defects in the matrix (Figure 5). Therefore, the compressive strength of the UHPC matrix increased when the W/C increased from 0.16 to 0.17 for the CGS-0.8 series.

Under the same W/C, the compressive strength decreased with the increase in the A/C of the UHPC matrix. However, with the increase in the W/C, the difference between the CGS-0.7 and CGS-0.8 systems in terms of compressive strength decreased gradually. For example, the compressive strength difference between the CGS-0.7 and CGS-0.8 systems decreased from 14.2 MPa to 0.2 MPa when the W/C increased from 0.16 to 0.19. When the W/C ranged from 0.16 to 0.19, the compressive strength of the UHPC matrix for the CGS system was 1.5–6.6 MPa higher than that of the CQS system since the GBFS with higher reactivity could produce more hydration products and resulted in a denser microstructure of the UHPC matrix [31,32].

### 4.2. The Flexural Strength of the UHPC Matrix

**Figure 6:**
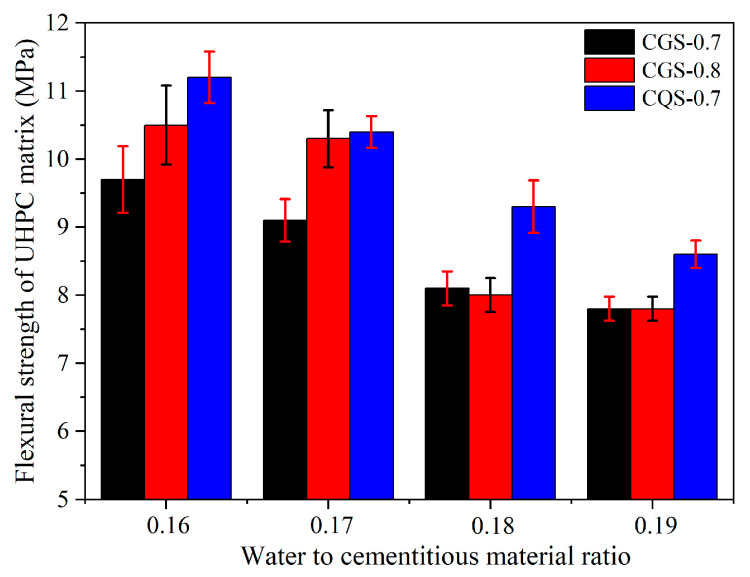
The flexural strength of the UHPC matrix.

As shown in Figure 6, the flexural strength of all UHPC matrices decreased with the increase in the W/C. When the A/C increased from 0.7 to 0.8, the flexural strength of the UHPC matrix in the CGS series increased by 8.2% and 13.2% for the W/C at 0.16 and 0.17, respectively. In contrast, the difference between the CGS-0.7 and CGS-0.8 systems in flexural strength was insignificant when the W/C was larger than 0.18. Compared with the flexural strength of UHPC for the CGS system, the CQS system had a higher flexural strength under the same W/C, which was inconsistent with the compressive strength of the UHPC matrix. Due to the higher hydration degree of GBFS than QP in UHPC, the shrinkage of UHPC using GBFS was greater, resulting in more microcracks and defects in the UHPC [33]. Therefore, the flexural strength of the UHPC matrix for the CGS system was lower than that of the CQS system.

In short, with the increase in the A/C from 0.7 to 0.8, the flexural strength of the UHPC matrix improved, while the compressive strength of the UHPC matrix decreased when the W/C ranged from 0.16 to 0.17. Compared with the UHPC matrix using QP, a UHPC matrix with higher compressive strength was obtained using GBFS. In addition, replacing granulated blast furnace slag (GBFS) with quartz powder (QP) could improve the flexural strength of the UHPC matrix without significantly reducing its compressive strength.

## 5. Mechanical Properties of the UHPC

According to the mechanical properties of the UHPC matrix, three UHPC matrices were selected to design the six UHPCs listed in Table 6. The slump flow, compressive strength, tensile strength, and flexural strength of UHPC were tested. The effects of matrix composition and steel fiber content on the mechanical properties of UHPC were discussed.

### 5.1. The Compressive Strength of the UHPC

**Figure 7:**
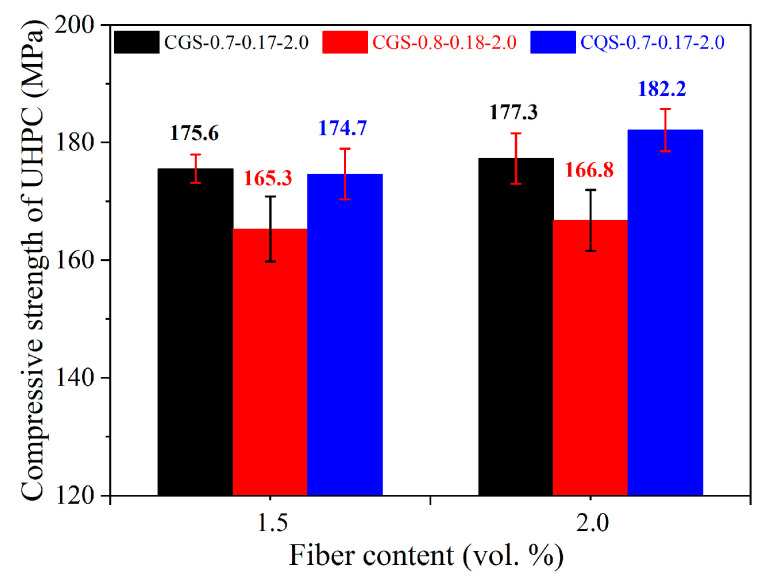
The compressive strength of the UHPC.

As shown in Figure 7, the compressive strength of UHPC gradually increased with the steel fiber volume fraction increase. Combined with Figure 4, the UHPC in the CQS-0.7-0.17 system showed the largest compressive strength increase when the steel fiber volume fraction increased from 0% to 2.0%. Under the same steel fiber fraction, the compression strength of the UHPC in the CGS-0.7-0.17 system was higher than that in the CGS-0.8-0.18 system, which was consistent with the findings in Section 4.1. When steel fiber was not used, the compressive strength of the UHPC matrix was reduced by replacing GBFS with QP. However, the compressive strength of the UHPC matrix was improved slightly by replacing GBFS with QP when the volume fraction of steel fiber was 2.0%.

### 5.2. The Tensile Strength of the UHPC

**Figure 8:**
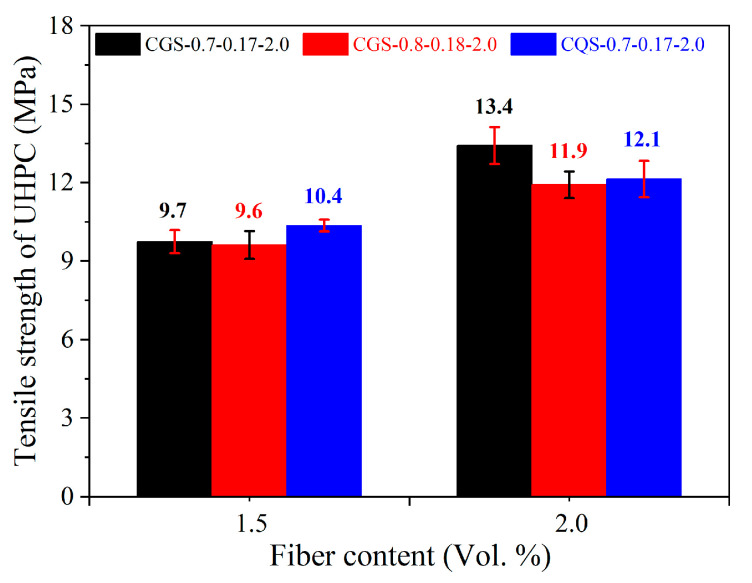
The tensile strength of the UHPC.

Compared with the compressive strength of UHPC, the tensile strength of UHPC increased significantly with the fiber volume fraction (Figure 8). When the steel fiber volume fraction rose from 1.5% to 2.0%, the tensile strength of the UHPCs increased by 16.3–38.1%. The difference between the CGS-0.7-0.17 and CQS-0.7-0.17 systems in terms of tensile strength was insignificant when the steel fiber with a volume fraction of 1.5% was used, while the tensile strength of the former was 10.7% higher than that of the latter when the volume fraction of steel fiber was 2.0%. The phenomenon can be attributed to the fact that GBFS with higher hydration activity generated more hydration products on the fiber surface and strengthened the fiber–matrix interface [34]. Although the shrinkage and initial microcracks of UHPC increased when GBFS was used, the steel fiber could effectively inhibit the generation of microcracks and the expansion of macroscopic cracks [13], resulting in the higher tensile strength of the UHPC.

### 5.3. The Flexural Strength of the UHPC

**Figure 9:**
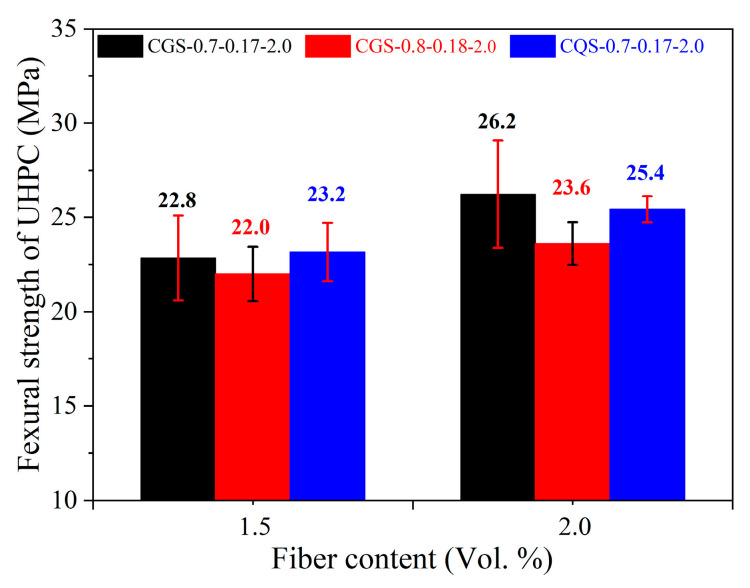
The flexural strength of the UHPC.

When the volume fraction of steel fiber increased from 1.5% to 2.0%, the UHPC in the CGS-0.7-0.17 system showed the highest increase in flexural strength, as shown in Figure 9. Under the same steel fiber fraction, the flexural strength of UHPC in the CGS-0.7-0.17 system was higher than that of the CGS-0.8-0.18 system, which was consistent with the flexural strength of the UHPC matrix. Combined with Figure 6, it can be seen that replacing the GBFS with QP enhanced the flexural strength of the UHPC matrix, while the flexural strength of the UHPC in the CGS-0.7-0.17 system was the highest when the fiber fraction was 2.0%. It can be inferred that an increase in steel fiber dosage may promote a more significant enhancement of GBFS on the flexural strength of UHPC.

In conclusion, the tensile strength of the UHPC showed the highest increase compared with the compressive and flexural strength of the UHPC when the steel fiber volume fraction increased from 1.5% to 2.0%. Under the same W/C and A/C, when the volume fraction of steel fiber increased to 2.0%, the compressive strength of UHPC using QP increased the most, while the tensile and flexural strength of UHPC using GBFS increased the most. At the steel fiber volume fraction of 1.5%, similar mechanical properties could be obtained by replacing GBFS using QP, indicating that inert fillers can be used to replace the supplementary cementitious materials whose particle size is between cement and silica fume.

## 6. Durability of the UHPC

### 6.1. Freezing–Thawing Resistance

**Figure 10:**
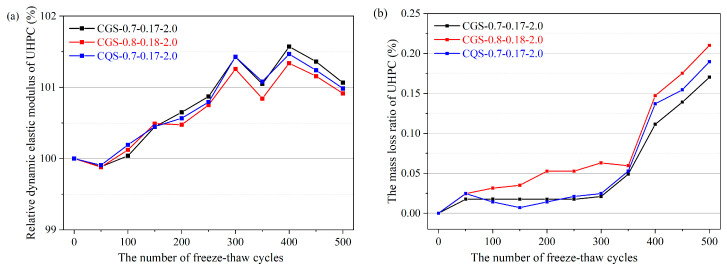
The freezing–thawing resistance of UHPC under different numbers of freeze–thaw cycles: (**a**) relative dynamic elastic modulus; (**b**) mass loss ratio.

As shown in Figure 10a, the relative dynamic elastic modulus of the UHPCs decreased slightly after 50 cycles of freezing and thawing. However, with the further increase in the number of freeze–thaw cycles, the relative dynamic elastic modulus of the UHPCs increased somewhat. When the number of freeze–thaw cycles reached 500, the relative dynamic elastic moduli of the UHPCs were 101.1%, 100.9%, and 101.0% for CGS-0.7-0.17-2.0, CGS-0.8-0.18-2.0, and CQS-0.7-0.17-2.0, respectively. This phenomenon can be attributed to the fact that the microstructure of UHPC is very dense and consequently has a low permeability [35,36]. In addition, the water content in UHPC is meager due to its low W/C. Thus, there are a large number of unhydrated cementitious material particles [37,38]. With the continuous hydration reaction, the microstructure of UHPC becomes denser during the testing process, resulting in a slow increase in the relative dynamic elastic modulus of UHPC [39].

**Figure 11:**
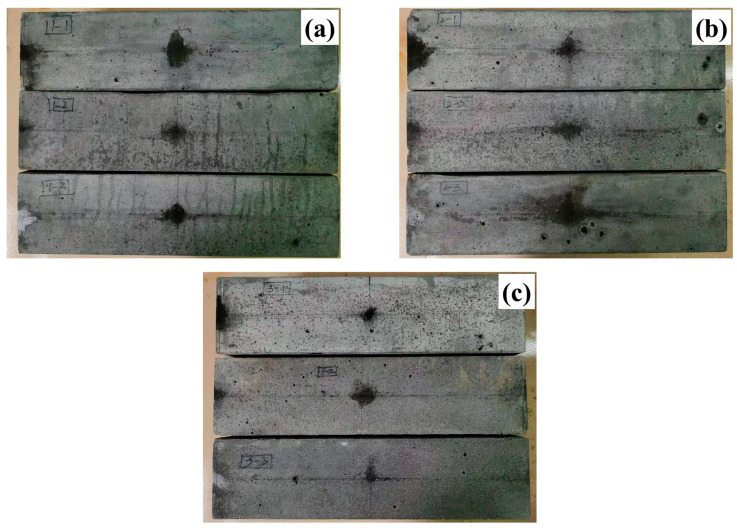
The surface of the UHPC after 500 freeze–thaw cycles: (**a**) CGS-0.7-0.17-2.0; (**b**) CGS-0.8-0.18-2.0; (**c**) CQS-0.7-0.17-2.0.

Figure 10b shows that the mass loss ratio of the UHPCs gradually increased with the increase in the number of freeze–thaw cycles. Under the same number of freeze–thaw cycles, the mass loss ratio of CGS-0.8-0.18-2.0 was the largest, while the mass loss ratios of CGS-0.7-0.17-2.0 and CQS-0.7-0.17-2.0 were similar. For instance, after 500 freeze–thaw cycles, the mass loss ratios of CGS-0.7-0.17-2.0, CGS-0.8-0.18-2.0, and CGS-0.7-0.17-2.0 were 0.170, 0.210, and 0.190, respectively. The freezing–thawing resistance of UHPCs can also be assessed based on the surface damage of UHPC. There were a few aggregates and fibers exposed on the surface and no obvious spalling for CGS-0.7-0.17-2.0 and CQS-0.7-0.17-2.0 after 500 freeze–thaw cycles, as shown in Figure 11a,c. In contrast, a few instances of spalling were found on the surface of CGS-0.8-0.18-2.0, as demonstrated in Figure 11b, from which it can be inferred that the better freezing–thawing resistances of CGS-0.7-0.17-2.0 and CQS-0.7-0.17-2.0 were because of the denser microstructure due to the lower W/C.

### 6.2. Sulfate Attack Resistance

**Figure 12:**
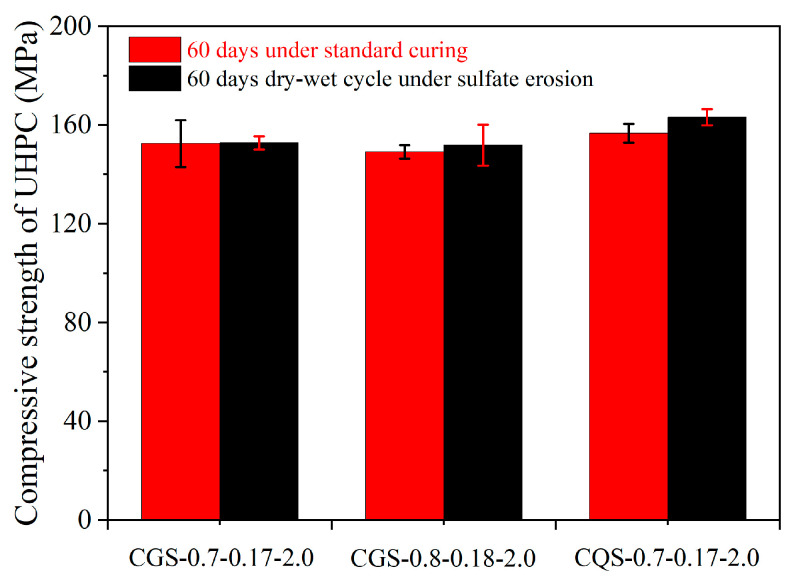
The compressive strength of the UHPC after standard curing or dry–wet cycles under sulfate erosion.

The compressive strength of UHPC after standard curing or dry–wet cycles under sulfate erosion is shown in Figure 12. The compressive strengths of UHPCs cured at 60 days under standard curing conditions were 152.6 MPa, 149.2 MPa, and 156.6 MPa for CGS-0.7-0.17-2.0, CGS-0.8-0.18-2.0, and CGS-0.7-0.17-2.0, respectively. Compared with the UHPC cured under standard curing conditions, the compressive strengths of UHPCs after 60 days of sulfate erosion under the dry–wet cycle were increased by 0.2%, 1.8%, and 4.2%, respectively. This phenomenon was similar to the relative elastic modulus of UHPC in the freezing–thawing test. It can be attributed that the permeability of UHPC was very low, and the compressive strength was further increased due to the heating process during the dry–wet cycles [40].

### 6.3. Chloride Penetration Resistance

The chloride diffusion coefficients were 0.88 × 10^−14^ m^2^/s, 0.11 × 10^−14^ m^2^/s, and 2.91 × 10^−14^ m^2^/s for CGS-0.7-0.17-2.0, CGS-0.8-0.18-2.0, and CGS-0.7-0.17-2.0, respectively, indicating that the designed UHPC had excellent chloride penetration resistance. Compared with CGS-0.7-0.17-2.0 and CQS-0.7-0.17-2.0, the W/C and A/C of CGS-0.8-0.18-2.0 were higher, from which it can be inferred that the better chloride penetration resistance could be achieved by increasing the A/C of the UHPC.

### 6.4. Carbonation Resistance

After 28 days of carbonation, the carbonization depth of all the UHPCs was 0 mm, indicating that all the designed UHPCs had excellent carbonization resistance.

## 7. Conclusions

Considering the effect of a water film covering the particle surfaces on particle packing, a method for UHPC mixture design was proposed, which employed the Fuller distribution mode. Conclusions that can be drawn from this study are summarized as follows:

(1) The UHPC matrix with a high solid concentration was designed based on the modified Fuller distribution mode. The solid concentration, slump flow, and compressive strength of the UHPC matrix reached 77.1 vol.%, 810 mm, and 162.0 MPa, respectively, when UHPC with a W/C of 0.16 was designed.

(2) With the increase in the A/C from 0.7 to 0.8, the flexural strength of the UHPC matrix improved, while the compressive strength of the UHPC matrix reduced when the W/C ranged from 0.16 to 0.17. Replacing granulated blast furnace slag (GBFS) with quartz powder (QP) could improve the flexural strength of the UHPC matrix without significantly reducing its compressive strength.

(3) When the steel fiber with a volume fraction of 1.5% was used, the slump flow, compressive strength, tensile strength, and flexural strength of the designed UHPC reached 740 mm, 175.6 MPa, 9.7 MPa, and 22.8 MPa, respectively.

(4) By replacing GBFS with QP, similar mechanical properties of UHPC were obtained. Therefore, inert fillers can be used to replace the supplementary cementitious materials whose particle size is between cement and silica fume for UHPC design.

(5) After 500 freeze–thaw cycles or 60 dry–wet cycles under sulfate erosion, the mechanical properties did not deteriorate. The chloride diffusion coefficients in the UHPCs were lower than 3.0 × 10^−14^ m^2^/s and the carbonation depth of each UHPC was 0 mm after carbonization for 28 days.

(6) The designed UHPCs presented ideal workability, mechanical properties, and durability, demonstrating the validity of the method proposed for UHPC design.

## Figures and Tables

**Table 1:** Chemical compositions of the materials used.

Material	Chemical Composition (%)									
	SiO_2_	Al_2_O_3_	Fe_2_O_3_	CaO	MgO	K_2_O	Na_2_O	SO_3_	Other	LOI ^a^
Cement	21.60	4.35	2.95	63.81	1.76	0.51	0.16	2.06	1.61	1.19
GBFS	20.37	4.45	3.49	64.79	0.88	0.64	0.08	2.70	1.46	1.14
QP	96.72	0.69	0.93	0.14	0.07	0.24	0.02	-	0.42	0.77
SF	98.07	-	0.12	0.51	0.31	0.53	0.14	0.12	0.19	0.01

^a^ LOI, loss on ignition; -, undetected.

**Table 2:** Physical properties and particle size statistics of the materials used.

Material	Specific Density (g/cm^3^)	Water Absorption (%)	Moisture Content (%)	Fraction (μm)	D_50_ (μm)
Cement	3.14	-	-	1.7–152.4	15.43
GBFS	2.80	-	-	1.5–51.5	8.82
QP	2.65	-	-	2.0–29.9	8.70
SF	2.20	-	-	0.1–0.5	0.25
Sand I	2.65	0.411	0.106	88.6–1019.5	394.24
Sand II	2.65	0.489	0.092	88.6–1754.6	557.81

**Table 3:** Mixture proportions of the cementitious materials.

Series	Cement (vol.%)	Silica Fume (vol.%)	Quartz Powder (vol.%)	Granulated Blast Furnace Slag (vol.%)
CGS	64.2	10.3	-	25.5
CQS	64.8	12.0	23.2	-

**Table 4:** Mixture proportions of solid particle mixtures and the solid concentration of the UHPC matrices.

Sample ID ^a^	Cementitious Materials (kg/m^3^)				Aggregates (kg/m^3^)		Water (kg/m^3^)	SP (kg/m^3^)	Solid Concentration (vol.%)
	Cement	GBFS	QP	SF	Sand I	Sand II			
CGS-0.7	893.2	312.6	-	98.3	228.6	684.3	194.2	21.5	77.14
CGS-0.8	850.3	297.6	-	93.5	248.7	744.4	182.0	24.8	78.40
CQS-0.7	905.7	-	271.7	117.7	227.0	679.5	192.5	22.0	76.86

^a^ Sample ID comprises the system of cementitious materials and the A/C.

**Table 5:** Mixture proportions and the slump flow of the UHPC matrices.

Series	Sample ID ^a^	Cementitious Materials (kg/m^3^)				Aggregates (kg/m^3^)		Water (kg/m^3^)	SP (kg/m^3^)	Slump Flow (mm)
		Cement	GBFS	QP	SF	Sand I	Sand II			
CGS-0.7	CGS-0.7-0.16	893.2	312.6	-	98.3	228.6	684.3	194.2	21.5	810
	CGS-0.7-0.17	883.0	309.1	-	97.1	226.0	676.5	208.4	16.1	815
	CGS-0.7-0.18	872.4	305.3	-	96.0	223.3	668.3	220.3	13.4	765
	CGS-0.7-0.19	861.6	301.6	-	94.8	220.5	660.0	230.6	12.6	785
CGS-0.8	CGS-0.8-0.16	850.3	297.6	-	93.5	248.7	744.4	182.0	24.8	725
	CGS-0.8-0.17	841.2	294.4	-	92.5	246.0	736.5	196.0	19.0	810
	CGS-0.8-0.18	832.3	291.3	-	91.5	243.4	728.6	209.8	13.4	775
	CGS-0.8-0.19	822.5	287.9	-	90.5	240.6	720.1	220.1	12.0	795
CQS-0.7	CQS-0.7-0.16	905.7	-	271.7	117.7	227.0	679.5	192.5	22.0	790
	CQS-0.7-0.17	895.5	-	268.7	116.4	224.5	671.9	207.0	16.0	810
	CQS-0.7-0.18	884.7	-	265.4	115.0	221.8	663.8	218.4	13.9	755
	CQS-0.7-0.19	873.9	-	262.2	113.6	219.1	655.7	229.1	12.5	765

^a^ Sample ID comprises the system of cementitious materials, A/C, and W/C.

**Table 6:** Mixture proportions and the slump flow of the UHPC.

Sample ID ^a^	Cementitious Materials (kg/m^3^)				Aggregates (kg/m^3^)		Water (kg/m^3^)	SP (kg/m^3^)	Steel Fiber (kg/m^3^)	Slump Flow (mm)
	Cement	GBFS	QP	SF	Sand I	Sand II				
CGS-0.7-0.17-1.5	869.8	304.4	-	95.7	222.6	666.3	205.2	15.9	117.0	740
CGS-0.7-0.17-2.0	865.4	302.9	-	95.2	221.5	662.9	204.2	15.8	156.0	670
CGS-0.8-0.18-1.5	819.8	286.9	-	90.2	239.8	717.7	206.6	13.2	117.0	615
CGS-0.8-0.18-2.0	815.6	285.5	-	89.7	238.6	714.1	205.6	13.1	156.0	585
CQS-0.7-0.17-1.5	882.1	-	264.6	114.7	221.1	661.9	203.9	15.8	117.0	695
CQS-0.7-0.17-2.0	877.6	-	263.3	114.1	220.0	658.5	202.8	15.7	156.0	645

^a^ Sample ID comprises the system of cementitious materials, A/C, W/C, and fiber content.

## Data Availability

The general data are included in this article. The original research data are available from the corresponding author upon reasonable request.
